# The use of bibliometrics in nursing science: Topics, data sources and contributions to research and practice

**DOI:** 10.1002/nop2.70036

**Published:** 2024-09-16

**Authors:** Belén Mezquita, Cristina Alfonso‐Arias, Patricia Martínez‐Jaimez, Ángel Borrego

**Affiliations:** ^1^ Departament de Ciències Bàsiques Universitat Internacional de Catalunya Sant Cugat del Vallès Spain; ^2^ Departament d'Infermeria Universitat Internacional de Catalunya Sant Cugat del Vallès Spain; ^3^ Facultat d'Informació i Mitjans Audiovisuals Universitat de Barcelona Barcelona Spain

**Keywords:** bibliographic databases, bibliometrics, citation analysis, citation indexes, nursing, scholarly journals, scholarly literature

## Abstract

**Aim:**

To describe the use of bibliometrics in nursing and assess their contribution to research and practice.

**Design:**

A content analysis was conducted of topics, data sources and applications of bibliometrics in nursing research articles.

**Methods:**

The study universe included 129 bibliometric articles on nursing retrieved from Scopus. A content analysis was performed to identify the purposes and topics of the articles, the sources employed to collect the data, the time frames covered, the amounts of records surveyed, and the features of the nursing literature analysed in bibliometric papers.

**Results:**

Nursing bibliometric research revolves around six key areas: global descriptions of the nursing literature, literature on specific nursing research topics, nursing education, nursing profession, nursing research using a certain framework or method, and nursing literature published in a country or region. Studies rely on three types of sources to retrieve the surveyed literature: bibliographic databases, sets of disciplinary journals and samples of documents. Bibliometrics can be employed to advance nursing research (identification of research gaps, establishment of research agendas, assessment of methodological approaches, etc.) and practice (identification of professional competences, categorisation of professional tasks, recognition of educational improvements, etc.), suggesting new avenues for researchers who aim to conduct further bibliometric research in the field. Further research is needed to assess the coverage of the nursing literature by new bibliographic data sources and to explore unaddressed topics such as gender imbalance in authorship.

## 1 INTRODUCTION

Bibliometrics consists of the quantitative analysis of scholarly publications. It can be used to track the growth of a particular area of research over time and to analyse literature features such as authorship, collaboration patterns or identification of core journals in a discipline. Bibliometric analysis is often used to assess the research output of individuals, institutions and countries based on citation analysis. In addition, bibliometric studies can be useful to unveil research gaps in a field, pinpoint conflicting findings, disclose underexplored areas or reveal research biases such as the presence of overrepresented or underrepresented populations. Overall, bibliometric studies provide valuable insights into the patterns and trends of scholarly communication and research, and can be used to inform research policy, funding decisions and strategic planning (Thompson & Walker, 2015).

Cant et al. (2022) recently described bibliometrics as “an emerging science in nursing.” However, the use of bibliometrics to describe and assess the nursing literature has bloomed over the past two decades. Within this context, it is crucial to perform empirical assessments of the use of bibliometrics in nursing to identify how bibliometrics can improve our understanding of the field and to provide a solid foundation for researchers who wish to conduct further bibliometric studies in the discipline.

The contribution of this paper is twofold. First, it describes and assess the use of bibliometrics in nursing research literature. The purpose is to ascertain how bibliometric studies contribute to advance theory and practice in nursing research, identifying the purposes and topics of bibliometric articles in nursing, the sources employed to collect the data, the time frames covered, the amounts of records surveyed, and the features of the nursing literature analysed in bibliometric papers. Second, it offers guidelines to help nursing scholars to conduct bibliometric studies in the field by using the appropriate techniques in each instance according to their purpose. In short, we aim to help researchers understand bibliometrics and its usefulness to improve our understanding of nursing science and how to use bibliometric techniques meaningfully and rigorously in the discipline.

### 1.1 Background

Bibliometrics aims to describe and map the scientific knowledge in a discipline by making sense of large volumes of bibliographic data. It can be useful to obtain an overview of a discipline, identify knowledge gaps and derive novel ideas for investigation (Donthu et al., 2021, p. 285). Bibliometrics encompass two categories of analysis: performance analysis and science mapping. The first examines the participation of research contributors (authors, institutions, journals, etc.) to a field, including publication and citation analysis. Science mapping focuses on the relationships between research contributors making use of techniques such as co‐authorship, co‐citations or co‐word analysis. The combination of both approaches is useful to describe the intellectual structure of a research field (Mukherjee et al., 2022).

In contrast to systematic reviews and meta‐analysis, bibliometric studies summarize the intellectual structure of a field by analysing the relationships between different research contributors. Rather than narrative synthesis of the content of individual articles based on manual analyses, bibliometric reviews provide quantitative measures and visualizations of large samples of papers that provide insights into the structure and evolution of the scholarly literature within a particular field.

The increasing interest in bibliometric studies within the health sciences has prompted the development of two recent sets of guidelines aimed at enhancing the reporting quality of such studies. Koo and Lin (2023) introduced the Preferred Reporting Items for Bibliometric Analysis (PRIBA) guidelines, comprising 25 items adapted from the PRISMA framework. Through an evaluation against the top 100 bibliometric studies in health and medicine from 2019 to 2021, these guidelines underscored the pressing need for improved reporting standards in bibliometric research. In parallel, Montazeri et al. (2023) formulated the Guideline for Reporting Bibliometric Reviews of the Biomedical Literature (BIBLIO), encompassing 20 items derived from a literature review and consensus among a panel of experts.

Few studies have discussed so far the usefulness of bibliometrics to improve our understanding of nursing science. In one of the first conceptual papers on the topic, Smith and Hazelton (2008) provided an overview of citation‐based research in nursing to map the core journals in the field and assess nursing research. Subsequently, Smith and Hazelton (2011) stressed their support for the use of bibliometrics and suggested that it should be included in nursing curricula. Later, Smith and Watson (2016) stated that bibliometric competencies should be expanded to altmetrics and social media.

Using a hands‐on approach, Alfonzo et al. (2014) outlined the basics of bibliometrics, the main steps in conducting a bibliometric study, features of bibliometric software and an example of applications with a small corpus in nursing research. In a similar fashion, Davidson et al. (2014) summarized the strengths and limitations of bibliometrics for mapping and assessing research performance in nursing, including webometric indicators such as the number of downloads or Twitter mentions.

Kokol and Vošner (2018) conducted the most extensive analysis of the application of bibliometrics in nursing to date. They analysed the historical roots of bibliometrics in nursing, the most productive countries, institutions and journals, and the most prolific themes in the application of bibliometrics in nursing. Their results showed a positive trend in literature production spread through all continents. Thematic analysis showed that applications of bibliometrics in nursing included descriptive analysis, research evaluation, content analysis, citation analysis and trend analysis.

### 1.2 Aims

In this study, we aim to provide additional insights of how bibliometrics is being used in nursing research. The purpose is to describe and assess the current state of bibliometrics in the field and to provide a solid basis for researchers who aim to conduct further bibliometric research in the discipline. The study is underpinned by the following research questions:

1. What are the topics of nursing bibliometric studies?
2. Which data sources, time frames and document populations are employed in nursing bibliometric research?
3. What bibliometric approaches are used to analyse nursing science?
4. What are the contributions of bibliometric studies to advance theory and practice in nursing research?

## 2 METHODS

### 2.1 Design

A content analysis of nursing bibliometric papers was conducted to identify the purposes and topics of the studies, the sources employed to gather the data, and the features of the literature analysed in the papers.

### 2.2 Data collection

On 10 February 2023, we retrieved from Scopus the articles published up to 2022 that included the terms “bibliomet*” and “nurs*” in the title of the document. The search was not limited by document type, language or any other criteria.

We retrieved 143 records. Since the purpose of the study was to analyse the practical applications of bibliometrics in nursing, five conceptual papers on the topic were removed from the analysis. Nine additional papers were removed because: (a) they were in Chinese (four records) or German (one record), languages that we do not understand; (b) they were not available online in full text (three records); or (c) they did not offer original data, but a comment on another bibliometric study that was already included in the sample (one record). In the end, 129 articles were analysed.

### 2.3 Data analysis

The full text of the 129 articles was downloaded. Each article was screened to collect information on the aim of the study, the data sources employed and the analysis conducted.

The articles had been published over the course of two decades, from 2001 to 2022, with a clear growing trend: 64 articles (50%) had been published in the last 3 years considered, from 2020 to 2022. The articles had been published in 62 journals, although five sources concentrated 29% of the literature, each publishing more than five nursing bibliometric studies: *Journal of Advanced Nursing* (12 articles), *International Journal of Nursing Practice* (7), *Journal of Nursing Management* (7), *Journal of Nursing Scholarship* (6) and *Texto e Contexto Enfermagem* (6).

### 2.4 Data availability

The 129 articles analysed are listed in the Annex. They are referred to throughout the article by means of numbers in brackets. In addition, the data resulting from this research are freely available in comma‐separated values (CSV) format (Borrego & Mezquita, 2023). For each article, the dataset includes the topic of the study, the data source employed to gather the literature, the time frame, the number of records considered, and whether the article analysed 14 variables.

## 3 RESULTS

### 3.1 Topics of nursing bibliometric research

**FIGURE 1:**
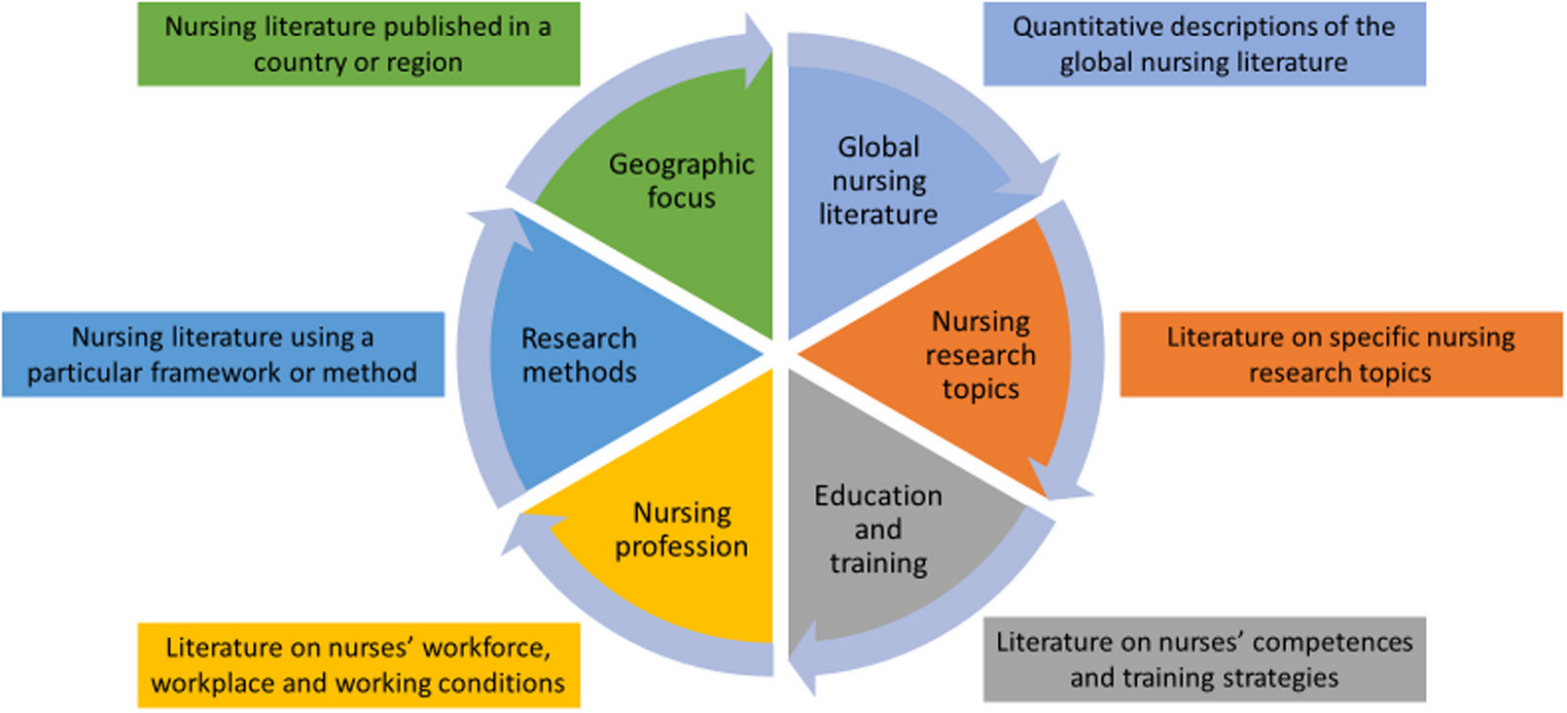
Topics of nursing bibliometric research.

Nursing bibliometric research can be broadly classified into six categories (Figure 1). First, 26 studies (20%) aimed to describe the global landscape of nursing scholarly literature. Second, 41 studies (32%) took a narrower approach to focus on the literature on a specific nursing research topic. Third, 23 studies (18%) analysed the literature on nursing education and training. Fourth, 20 studies (16%) explored the literature on the nursing profession, i.e., the workforce, workplace and working conditions. Fifth, 7 studies (5%) dealt with the features of nursing research that used a certain theoretical framework or research method. Finally, 12 studies (9%) analysed the nursing scholarly output published in a particular country or region. Some of the studies classified in the first five categories also had a geographic focus (e.g. a bibliometric analysis of the literature on a nursing topic published in a particular country). For the purposes of this paper, we classified these articles in the first five categories rather than in the sixth.

#### 3.1.1 Bibliometric studies on global nursing literature

The studies that took a broader approach aimed to provide an overview of nursing scholarly literature published in a certain time frame. For instance, Wang et al. [109] performed a global assessment of nursing research indexed in the Web of Science from 2009 to 2020. The results showed a positive publication trend of nursing papers, although restrained by low research funding, regionally centred research activity and limited international collaboration in developing regions.

Instead of searching a database to retrieve the literature in the field, some nursing bibliometric studies focused on the outputs published in a set of disciplinary journals. For example, Giménez‐Espert and Prado‐Gascó [39] analysed the outputs published between 2012 and 2017 in the six most reputable nursing journals according to their impact factor. Their results allowed an assessment of the state of nursing research by revealing the most popular authors, institutions and topics.

Alternatively, some studies focused on the bibliometric indicators of disciplinary journals. Thus, Smith [97] performed a longitudinal analysis of the evolution between 1977 and 2008 of seven nursing journals, especially their impact factors, which showed a sustained increase in the number of citations received. Similarly, Avena and Barbosa [5] compared bibliometric indicators provided by six databases for a set of seven Brazilian and seven international nursing journals between 2012 and 2014.

Finally, several case studies focused on particular nursing journals. The literature included studies on the Brazilian nursing journal *Referência* [88], the *Journal of Advanced Nursing* [121], the *International Journal of Nursing Studies* [50], *The Canadian Nurse* [71], the *Philippine Journal of Nursing* [106], the *Journal for Nurse Practitioners* [108] and the *Journal of Nursing Management* [118].

#### 3.1.2 Bibliometric studies on specific nursing research topics

Most nursing bibliometric studies analysed the literature on a particular issue. The range of nursing research topics explored included artificial intelligence in nursing [94], cardiovascular nursing [15], cirrhosis nursing [42], COVID‐19 nursing [22; 84; 123], disaster nursing [76; 125], family nursing [46], genomic nursing [113], geriatric and gerontologic nursing [38; 79], HIV/AIDS nursing [29], intensive and coronary care nursing [67], mental health nursing [61], military nursing [25], multiple sclerosis nursing [119], nursing and ageing [90], nursing caregiving [33], oncologic nursing [124] and palliative care nursing [37].

In addition to bibliometric studies designed to quantify the literature on a particular topic, some studies employed bibliometrics to explore other features of the nursing literature. This suggests that there are additional applications of bibliometrics in nursing. Some bibliometric studies described the literature on research utilization in nursing [35; 93]. Another study used bibliometrics to measure the use in the literature of five nursing terminology sets [4]. Yet another approach consisted of exploring the comprehensiveness of systematic reviews in four guidelines for preventing in‐patient falls by comparing their references lists with all the available literature [21].

#### 3.1.3 Bibliometric studies on nursing education and training

Papers classified in this category revolved around two main issues: nurses' competences and training strategies to improve nurses' education. Blažun Vošner, Kokol and Vošner [14] analysed the nursing competences research literature indexed in Scopus between 1981 and 2012. Su, Hwang and Chang [99] analysed nursing core competencies research indexed in the Web of Science between 1997 and 2022. Specific studies focused on the literature on certain competences of nurses, such as clinical reasoning [44], informatics [13; 57; 58; 62; 64; 65] and leadership [53; 81].

Regarding bibliometric studies on training strategies, some papers focused on the use of expressions of art in nursing education and care [36], robots [91], simulation [17; 111] or virtual simulation [18; 122].

#### 3.1.4 Bibliometric studies on the nursing profession

Another topic of interest for nursing bibliometric research was literature on the workforce of nurses, the workplace and working conditions. Focusing on nursing professional practice, we found bibliometric studies on nursing advocacy [10], dignity care [66], patient satisfaction [100], nurse residency programmes [31], nurse rounding [49] or the use of robots to assist nursing care [19].

Other bibliometric studies focused on nurses' working conditions. They explored literature on the burnout syndrome among nurses [28; 32; 72], conflicts in nursing [53], job insecurity [89], nursing as a career [12], nurses' turnover [70], nurses' wellbeing [51], workplace bullying [68] and workplace incivility [103].

#### 3.1.5 Bibliometric studies on nursing literature using a particular framework or research method

Some bibliometric papers focused on nursing literature that used a particular theoretical framework, research methodology or data collection technique. This category included bibliometric papers on nursing studies using action research [74], grounded theory [60], phenomenological approaches [75], qualitative methods [8; 77] and social representation theory [26].

#### 3.1.6 Bibliometric studies on nursing literature in a geographic area

Finally, some bibliometric studies focused on the nursing literature published in a particular country or region. There were studies on nursing research in the Arab region [27], Australia [112], China [86], Colombia [16; 41], Spain [85], Taiwan [47] and Turkey [34]. Although most of these papers used a case study approach, which focused on the literature published in a single country, one study [104] compared nursing research published in six mainly English‐speaking nations (Australia, Canada, Ireland, New Zealand, the United Kingdom and the United States). Another international study [59] correlated nursing literature production with country and health determinants, including life expectancy, gross domestic product, human development factor and gross national income.

### 3.2 Data sources in nursing bibliometric research

Nursing bibliometric studies relied on three types of sources to retrieve the surveyed literature. First, 95 studies (74%) analysed the literature retrieved through one or several bibliographic databases. Second, 21 studies (16%) focused on articles published in a set of disciplinary journals. Third, 13 studies (10%) surveyed documents that shared a particular feature, such as having been selected by a group of experts or being a particular type of document, usually a dissertation or theses.

The three approaches were not mutually exclusive. Thus, a study analysing the literature published in a set of journals could rely on a bibliographic database to retrieve the records employed in the analysis. For the purposes of this paper, we classified these articles as being based on a set of journals rather than on a database.

#### 3.2.1 Literature retrieved from bibliographic databases

The most common approach to data collection in nursing bibliometric studies was to retrieve the literature from one or several databases. Up to 39 sources were mentioned in the articles, with several studies combining two or more sources. The databases employed most frequently were the Web of Science (49 articles), Scopus (23), MEDLINE/PubMed (23) and the Cumulative Index to Nursing and Allied Health Literature (CINAHL; 11).

In addition to their prestige, the preference for Web of Science and Scopus is explained by the fact that they are citation indexes, i.e., they list the references cited in the articles that they cover. This allows data to be obtained on the references cited by an article and the citations it receives. These data are not available for other databases, which prevents citation analysis, unless data from several sources is compiled. For instance, in a bibliometric study on robotics in nursing, Carter‐Templeton et al. [19] used CINAHL to identify the literature. Afterwards, citation counts were collected via Google Scholar, Scopus and the Web of Science. Similarly, in their article on distinct nursing research, Nicoll et al. [82] asked journal editors to identify relevant articles and the author then collected citations to these articles from Scopus.

The combination of several databases in a single study allows researchers to assess their comprehensiveness. For instance, Scott et al. [93] concluded that, at the time of their analysis, CINAHL was more comprehensive than either MEDLINE or the Web of Science in covering references in the knowledge utilization field in nursing.

Since most nursing bibliometric studies focused on publications on a certain topic, the most usual approach to retrieve the literature was keyword searching. Nevertheless, other search strategies were applied when relevant. Thus, Huang, Ho and Chuang [47] searched the Web of Science for nursing papers published by authors based in Taiwan to analyse the scholarly literature published in the country.

Finally, some authors focused on the most cited articles according to a citation index. This was the case of bibliometric studies of the top 10% of cited papers in nursing published between 2008 and 2018 [129], or the 100 most cited articles on nursing student education published between 2000 and 2020 [20]. Both studies relied on citation metrics provided by the Web of Science.

Some studies relied exclusively on databases that provide bibliometric indicators. Thus, Smith [97] used the *Journal Citation Reports* to conduct a longitudinal analysis between 1977 and 2008 of impact factor trends among seven core journals in nursing. Santiago and Carlantonio [92] and Singh and Pandita [96] used bibliometric indicators provided by the *Scimago Journal and Country Rank* to measure nursing research output in the BRIC countries (Brazil, Russia, India and China) and worldwide, respectively. These databases provide information on the number of articles published and citations received by journals in a field but fail to include any information on individual articles.

#### 3.2.2 Literature published in a set of journals

Instead of retrieving records from literature databases, another option used in nursing bibliometric research was to analyse the articles published in a set of journals selected for their thematic orientation, their geographic origin or their citation impact. When the population of articles was large, some kind of sampling was applied. For instance, in their analysis of Australian nursing research between 1985 and 2010, Wiles, Olds and Williams [112] consistently sampled seven journals at 3‐month intervals every 5 years from the first year of publication to 2010.

Journals and databases can be combined to improve the comprehensiveness of data gathering. Thus, in their analysis of the United Kingdom nursing literature, Traynor et al. [105] initially identified all United Kingdom papers published in 23 nursing journals. These articles were supplemented with papers published in other “general” journals retrieved through a database, if they had one or more of a set of title keywords.

#### 3.2.3 Sets of documents

Some nursing bibliometric studies analysed samples of articles selected according to various criteria. This approach was employed, for instance, in two citation analyses of “distinct” nursing literature [82; 83] that required journal editors to submit articles from their journals representing “distinction” in nursing research, education or practice. Other nursing bibliometric studies focused on particular document types, such as theses and dissertations [95] or nursing guidelines [21].

In terms of time frames, on average, each bibliometric study covered 20.9 years (median = 17 years). Although some bibliometric studies went further back in time, most research was concentrated in the period between 2001 and 2015, with numerous studies taking the year 2000 as the starting point for their analysis.

In terms of size of the populations under study, the biggest samples were those of bibliometric studies based on sets of journals (average = 1693 records, median = 825), followed by studies based on bibliographic databases (average = 1329, median = 433) and those based on sets of documents (average = 159, median = 108). The smaller size of samples in studies based on literature searches is explained by the presence of studies that focused on specific research topics, with little scholarly output. Studies based on sets of documents frequently focused on a relatively small number of theses and dissertations.

### 3.3 Types of analysis in nursing bibliometric studies

**FIGURE 2:**
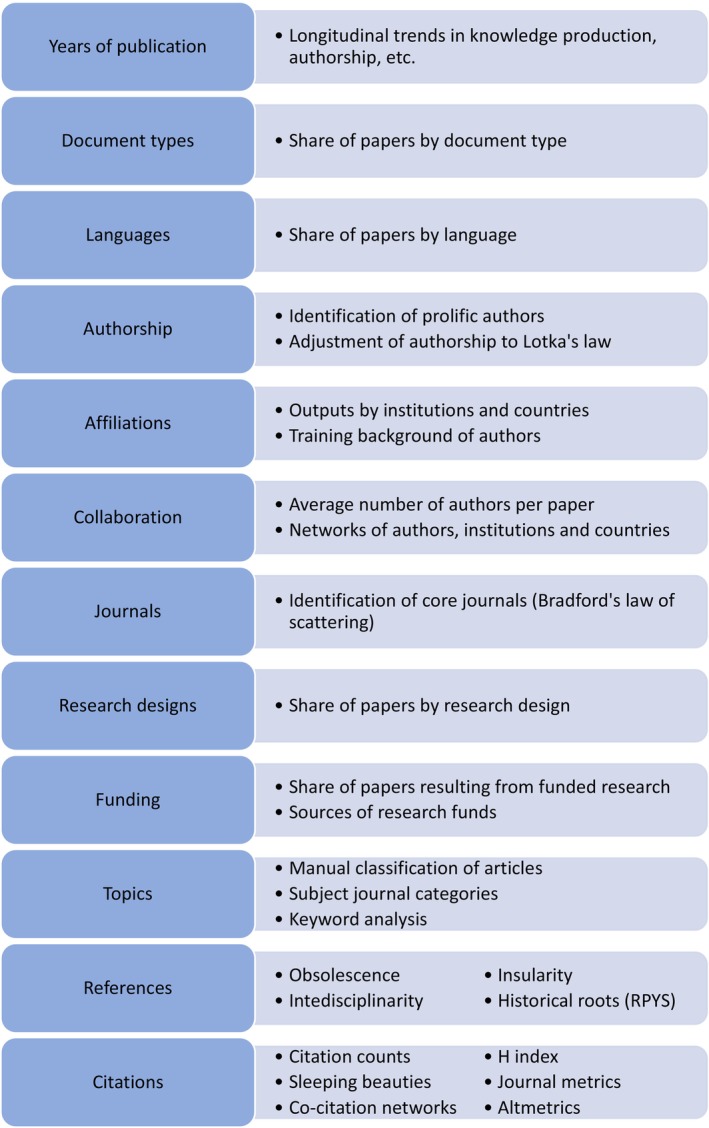
Types of analyses in nursing bibliometric research.

This section focuses on the features of the literature analysed in nursing bibliometric studies. Twelve variables were analysed in the articles surveyed, which revealed different applications of bibliometrics to study nursing science (Figure 2).

#### 3.3.1 Years of publication

The most common variable analysed in nursing bibliometric research was the year of publication of the surveyed documents. Most bibliometric studies (96 studies, 74%) included a longitudinal analysis of trends in knowledge production. Although the analysis was usually limited to counting the number of articles published per year, similar analyses can be applied to other variables. For instance, a case study of the *Journal of Advanced Nursing* [121] discussed the annual trends in number of authors, pages, institutions, references and citations per article.

Most studies revealed an upward trend in the volume of published articles, often interpreted as indicative of growing interest in the subject matter. While such a rise may indeed signify heightened interest, the proliferation of published articles may also be influenced by the expansion of databases used for data collection. The continual enlargement of journals indexed in Scopus and Web of Science contributes to an augmented retrieval of scholarly outputs across various topics.

#### 3.3.2 Document types

As discussed above, most nursing bibliometric studies relied on bibliographic databases to retrieve the literature. These databases mostly index scholarly journals, including different types of documents: articles, editorials, letters, reviews, etc. The classification of documents according to these categories was presented in some nursing bibliometric studies (25 studies, 19%). The analysis of document types can be combined with other variables of interest. For example, Anderson, Keenan and Jones [4] analysed to what extent five nursing terminology sets were used in different document types. Nevertheless, it is important to note that such analyses are often constrained by the database's coverage, which tends to prioritize the indexing of journal articles over other document types. This limitation may impact the comprehensiveness and scope of the analysis.

#### 3.3.3 Language of documents

As in the case of document types, this kind of analysis (11 studies, 9%) relied on the information provided by bibliographic databases. When conducting analyses of languages within the literature, it is crucial to consider that biases inherent in the database's coverage can significantly influence the outcomes. Put simply, the proportion of nursing literature available in various languages as retrieved from databases like Scopus and the Web of Science is contingent upon the language coverage provided by the database itself. Therefore, it may not accurately reflect the entirety of scholarly output in the field.

#### 3.3.4 Authorship

In the analysis of authorship (48 studies, 37%), the most frequent output delivered by nursing bibliometric papers was a list of the most prolific authors. Nevertheless, some studies [e.g., 2; 3; 35; 72] went one step further to analyse whether the distribution of authors followed Lotka's law of productivity. This law describes the frequency of publication by authors in any given field. It states that the number of authors publishing *n* papers is 1/n^2^ of those publishing one paper (Pao, 1985).

#### 3.3.5 Affiliations

The analysis of the affiliations of the authors of the literature allowed identification of the most productive institutions (64 studies, 50%) and the most productive countries (69 studies, 53%). In some instances, this analysis became the focus of the study. Thus, Kokol et al. [59] explored the relationship between nursing research literature production and country and health determinants. They concluded that gross domestic product, human development factor, and gross national income were related to nursing research literature productivity.

In addition to institutional affiliations, some nursing bibliometric studies analysed the education background of the authors who contributed to the literature. Thus, Ravelli et al. [90] analysed the training (e.g., nurses, doctors, pharmacists, odontologists, psychologists, etc.) of the authors of Latin‐American literature on nursing and ageing published between 2003 and 2008. In a similar fashion, Marcellus [71] analysed the longitudinal evolution of the presence of nurses, physicians and other health professionals among the authors in *The Canadian Nurse*.

#### 3.3.6 Collaboration

Research collaboration was explored through the analysis of co‐authorship (39 studies, 30%). The analysis can be purely numerical, i.e., calculating the average number of authors per paper, or can be aimed at identifying the networks of researchers, institutions and countries that publish jointly. For instance, Alcalá‐Albert and Parra‐González [3] built a network of co‐authors who frequently published together in the nursing outputs indexed until 2021 in the Web of Science, whereas Zhu et al. [129] built a network of co‐author institutions in their analysis of the top cited papers in nursing between 2008 and 2018.

In most studies, full counting was used for handling publications with multiple coauthors. However, proper field normalization requires fractional counting (Waltman & van Eck, 2015). These two approaches yield different results in co‐authorship networks and journal coupling networks, with fractional counting being preferable over full counting (Perianes‐Rodriguez et al., 2016).

#### 3.3.7 Journals

The core journals in the field can be identified through an analysis of journals that publish nursing literature. This type of analysis was present in 69 studies (53%). Beyond a list of journals sorted by the number of articles published, some researchers [e.g., 3; 35; 67; 68; 79] explored whether journals in their analyses followed Bradford's law of scattering. This law states that, if journals in a field are sorted by number of articles, they can be divided into a nucleus of journals that are more specifically devoted to the subject and several groups containing the same number of articles as the nucleus, when the number of journals in the nucleus and succeeding groups will be as 1:n:n^2^, where *n* is a multiplier (Desai et al., 2018). However, it is essential to consider that different operationalizations of the concept of “subject” can lead to significantly different lists of core journals (Nicolaisen & Hjørland, 2007).

#### 3.3.8 Research designs

Some nursing bibliometric papers (26 studies, 20%) analysed the methodological features of the articles surveyed. For example, in their longitudinal analysis of Australian nursing research, Wiles, Olds and Williams [112] verified an improvement in research designs, with an increasing use of higher research approaches and greater quantification in reporting results. Similarly, Chang et al. [20] analysed the chronological evolution in the statistical analyses employed in the top 100 most cited articles on nursing student education published between 2000 and 2020.

#### 3.3.9 Funding

Although it was not common (14 articles, 11%), some bibliometric studies reported findings on the funding of nursing research. For example, McVicar, Munn‐Giddings and Abu‐Helil [74] analysed the funding sources of nursing literature in the UK that used action research as a methodology. Huang et al. [46] explored institutions funding family nursing research using data from Web of Science.

In this kind of studies, it is necessary to bear in mind that coverage of funding information differs significantly among Scopus, Web of Science and PubMed for the same journals (Kokol, 2023; Kokol & Vošner, 2018). Consequently, the choice of a bibliographic database could potentially introduce bias into the results. To mitigate this bias, researchers aiming to retrieve funding information related to specific research topics or institutions should consider combining data from all three databases to obtain more complete information.

#### 3.3.10 Topics

Most nursing bibliometric studies (92 studies, 71%) aimed to identify the topics, themes and research hotspots in the literature. Three alternative methods were used for this purpose: manual classification of articles by topic, assignation of articles to the subject categories of journals in the database, and keyword analysis.

In the case of manual classification of articles, the subject categories may be built purposely by researchers, or may rely on existing classification schemes. Using the first approach, Scott et al. [93] assigned articles on research utilization in nursing to ten “domains” created by the authors. Zhang et al. [125] used the International Council of Nurses' Framework of Disaster Nursing Competencies to classify articles on the topic.

In other studies, the classification of articles by topic was not based on the direct examination of papers but on the databases' journal categories. For example, in their analysis of the literature on robots in nursing education, Romero, La Hoz and González [91] classified articles according to the subject areas employed by Scopus to classify the journals.

Finally, some authors used keyword analysis to identify the topics in the literature. Keywords were extracted from titles and abstracts or were provided by the database, such as the Medical Subject Heading in the case of PubMed. The analysis usually consisted of counting the frequency of keywords, to be depicted in word clouds, or identifying co‐occurrence networks of keywords, which were usually clustered and depicted using VOSviewer software (Van Eck & Waltman, 2010).

The selection of any of these approaches can significantly influence the outcomes. Journal classifications within databases may not always encapsulate the specific topics or themes explored within individual articles, particularly in multidisciplinary journals or those exploring emerging research areas. Moreover, these classification systems may lack the granularity necessary to distinguish between closely related topics and may encounter issues of inconsistencies.

Employing keywords extracted from titles and abstracts offers a more flexible and tailored method for defining topics and themes. This strategy allows for the inclusion of synonyms, related terms, and variations in spelling or terminology. Utilizing database‐provided keywords presents certain advantages in terms of consistency and comprehensiveness, given that most databases utilize controlled vocabularies and subject indexing to categorize publications. This can ensure consistency in topic identification across various studies. However, predefined keywords may not fully encompass the breadth and diversity of topics within a research area, nor do they necessarily reflect emerging or niche areas that have yet to be integrated into the database's subject indexing.

A combination of both methods may prove beneficial. By extracting keywords from titles and abstracts and cross‐referencing them with database‐provided keywords, researchers can ensure a comprehensive and consistent approach to topic identification.

#### References

3.3.11

References cited in papers were another feature of interest in nursing bibliometric research. Some articles focused exclusively on references cited in the literature to analyse their characteristics: years of publication, document types, languages, etc. The list of variables susceptible to be analysed in references is as long as the list of variables that can be analysed in the citing articles themselves.

The simplest analysis consisted of measuring the average number of cited references per document and whether there were any differences between, for instance, document types (e.g., whether research papers or clinical studies cite different numbers of sources). Some studies focused on references to particular types of documents. For example, Woods, Phillips and Dudash [115] analysed the references to grey literature (conference proceedings, news, theses and dissertations, etc.) in six nursing journals.

The Price index, that is, the percentage of references that are less than five years old, allowed an exploration of obsolescence of the literature [72]. In studies focusing on the literature published in a particular country, the share of references published in the same country as the surveyed articles allowed the “insularity” of the field to be measured [34; 85]. The interdisciplinarity of the discipline can also be analysed by measuring the share of nursing and non‐nursing references in the nursing literature [35]. Finally, the analysis of references cited in nursing guidelines can be a method to assess their comprehensiveness [21].

Reference Publication Year Spectroscopy (RPYS) is a bibliometric method that can be used to track the historical origins of research fields. RPYS plots the cumulative distribution of cited references of a publication set in terms of the referenced publication years. The peaks in the graph indicate specific publications which were cited frequently within the field (Marx et al., 2014). This method was applied to the global nursing literature [56], cardiovascular nursing [15], nursing informatics [13] or meta‐analysis approaches used in nursing research [55].

#### 3.3.12 Citations

As in the case of references, the citations received by an article may be analysed from multiple points of view: years of publication of citing documents, types of citing documents, citing authors, etc. Again, the simplest analysis consisted of counting the number of citations per paper. To make the analysis more meaningful, citation counts can be combined with other variables to determine, for instance, whether articles are cited more if they are written in international co‐authorship [47] or if they result from funded research [105].

A popular citation indicator at author level is the h index, which combines the productivity and citation impact of an author. The h‐index is defined as the maximum value of h such that the given author has published at least h papers that have each been cited at least h times (De Groote & Raszewski, 2012). Goode et al. [40] calculated the h index for nurses at the University of Colorado Hospital in their case study of the contribution of this institution to nursing scholarly literature. Similarly, Singh and Pandita [96] calculated the h index for the countries that contribute to nursing scholarly literature according to the *Scimago Journal and Country Rank*.

“Sleeping beauties” are publications that go unnoticed for a long time and then, almost suddenly, attract a lot of attention (Van Raan, 2004). Železnik, Blažun Vošner and Kokol [121] identified two sleeping beauties in their 40‐year analysis of the *Journal of Advanced Nursing*.

A possible way to establish associations between research agents is co‐citation, that is, when two articles, authors, journals, etc. are cited together. For instance, in their analysis of the nursing output published in six journals, Giménez‐Espert and Prado‐Gascó [39] built a network of co‐cited articles. Scott et al. [93] built a co‐citation map of the most cited authors in the field of nursing utilization to unveil the structure of the scientific community that works in the field. Guo, Lu and Tian [42] built a network of co‐cited journals in the field of cirrhosis nursing.

In their analysis of “distinct” nursing research, Nicoll et al. [82; 83] analysed three citation features of a set of articles selected by journal editors: persistence (rate of subsequent citations over time), reach (geographic distribution of subsequent citations) and dissemination (specialty of follow‐on citations represented as nursing or another discipline). In addition to persistence, read and dissemination, Waldrop, Carter‐Templeton and Nicoll [108] analysed altmetrics provided by PlumX (usage, captures, mentions and social media) in a case study of the *Journal for Nurse Practitioners*.

Finally, it should be noted that some of the bibliometric studies identified in this research focus on the bibliometric analysis of highly cited articles. When this approach is used, authors should consider the time lapse needed by articles to accumulate citations. The most cited articles in, say, the past 20 years are not necessarily representative of the more impactful research published in these two decades, since older articles have had more time to accrue citations and may introduce a bias in the sample. This should be acknowledged as a limitation in this kind of studies. Recent groundbreaking research might be overlooked if the analysis focuses solely on the most cited works. This can skew the understanding of current trends and developments in a field.

## 4 DISCUSSION

### 4.1 Contributions of bibliometric studies to advance nursing research and practice

Bibliometrics involves the quantitative analysis of scholarly publications. It is used to explore the features of nursing science has sharply increased in recent years. Nevertheless, bibliometrics is an umbrella term that encompasses a wide range of analyses. This study offers a detailed overview of the applications of bibliometrics in nursing research, which suggests new developments for researchers who aim to conduct further bibliometric research in the field.

Most nursing bibliometric studies combine performance analysis with science mapping to advance nursing research and practice. Most papers identify research leaders, collaboration patterns, influential journals, hot topics, research frontiers, etc. This information is useful to assess the maturity of a research topic. In some instances, limited amount of collaborative research and the repeated citation of a few references pinpoint towards the underdevelopment of research fields such as research utilization in nursing [35]. These observations are of interest to policy makers who can become aware of the need to diversify grant support to broaden the scope of nursing research in themes such as artificial intelligence [94] or in genomic nursing science [113].

The identification of research gaps and the establishment of research agendas are among the most usual contributions of bibliometric studies when applied to topics such as burnout among nursing professionals [32] or conflict in nursing [53]. Nursing bibliometric research focused on specific geographic areas can reveal the need of researchers in certain countries to investigate specific patient groups, diseases, treatments or skills for unresearched gaps with national relevance [104]. Even individual case studies focused on a single journal can reflect emerging scientific developments and evolving social values [71].

Bibliometric studies contribute to understanding the global direction of the field and specific research topics. For instance, a bibliometric study on leadership and care in nursing revealed that themes such as job satisfaction, teamwork and retention were researched more intensively whereas patient‐based and fundamental‐care themes, including patient safety, comfort, dignity or privacy were less studied [54]. Similarly, a keyword analysis of studies on workplace incivility in nursing revealed that horizontal/lateral violence and bullying were used interchangeably even though their contents and meaning are quite different [103].

Some bibliometric studies unveil different approaches to research in a certain topic. Keyword co‐occurrence analysis has proved useful to identify how different health sciences address the field of scope of practice, identifying areas where synthesis to find consolidated results may be possible [9]. Using a slightly different approach, bibliometric analysis of the literature on nursing competences allowed to identify different approaches to the topic of nursing competence in the practice‐oriented versus the educational literature [14]. In a similar fashion, bibliometric research can be useful to show the transferability potential of knowledge gained from nursing research to other health professions, as in the case of military nursing research [25].

From a methodological point of view, bibliometric studies are useful to assess the comprehensiveness of systematic reviews as illustrated by a comparative analysis of guidelines for preventing inpatient falls [21]. Low extensiveness revealed certain preferences in how the literature was selected, suggesting how to improve reviews in terms of methodological quality. Citation analysis can also be relevant to show the substantial use of grey literature and the need to apply this knowledge to instruction, research and practice [115].

Our results also illustrate how bibliometrics can improve nursing practice. Empirical evidence of correlation between literature production and well‐being and health determinants of countries can be useful to demonstrate that research is successfully translated into practice [59]. Similarly, a 20‐year bibliometric analysis of nursing research in Australia was useful to demonstrate the increased sophistication in the impact of nursing services on access to care [112].

RPSY is a viable approach to analyse the historical roots of knowledge development that can be useful to better understand current problems in the profession. For instance, it may help nurse practitioners to recognize ingrained cultural traditions and cultural bias, so they can become more culturally sensitive to people from other cultural environments [15]. In a similar fashion, a bibliometric analysis of research in the field of nursing management, religion and spirituality revealed the need that nurses are equipped to develop an understanding of the socio‐religion changes towards personal spiritual inquire and development [23].

Bibliometric methods are useful to improve our understanding of the profession, as evidenced by studies of the literature of robotics in nursing that allowed to identify and classify applications of robotics within patient care areas [19] or a proposal for a categorisation of variants of nurse rounding based on bibliometric data [49].

Bibliometric studies have revealed that nurses should be included in decision‐making policies, as illustrated by a bibliometric study on nurses as agents for achieving environmentally sustainable health systems [69].

Finally, bibliometric studies can enlighten how to improve nursing education, as exemplified by research that identified core competencies in critical thinking, complex problem solving or computation thinking [99]. Another bibliometric analysis of simulation in nursing education showed that psychiatric simulations and critical care simulation were core priorities in nursing education [111].

### 4.2 Methodological approaches in nursing bibliometric research

Our results show that the topics of nursing bibliometric research revolve around six main areas. Most studies aim to analyse the nursing scholarly literature, either globally or focusing on particular research topics. Other issues of interest for nursing bibliometric research are nursing education, nursing as a profession, nursing research using a particular research method and nursing literature published in a particular country or region. This landscape is consistent with the descriptions of the nursing research activity provided by Alcalá‐Albert and Parra‐González (2021) or Wang et al. (2022) who observed an upward trend in publications on topics such as nursing education and the high burden of care among nurses, resulting in stress and burnout syndrome.

Regarding data sources, most studies rely on bibliographic databases, either multidisciplinary citation indexes (the Web of Science and Scopus) or index and abstract databases in the health sciences (PubMed and CINAHL). Previous research has shown that Scopus offers better coverage than the Web of Science for reporting nursing publication metrics (Powell & Peterson, 2017). These sources are suitable for systematic reviews and meta‐analysis, offering effective and efficient search results with regards to precision, recall and reproducibility (Gusenbauer, 2022; Gusenbauer & Haddaway, 2020). Two studies referred to the use of Google Scholar as a data source, although research has shown its unreliability, as evidenced by its inability to provide consistent results for identical queries (Gusenbauer & Haddaway, 2020). There was no mention of new open bibliographic sources such as Crossref, Dimensions or OpenAlex that supplement traditional citation indexes in bibliometric studies (Chawla, 2022). Research suggests that these new databases have a similar or better coverage than traditional sources (Harzing, 2019; Martín‐Martín et al., 2021). Further studies are needed to confirm whether these sources may serve as good alternatives to Scopus and the Web of Science for literature reviews and citation analysis in nursing.

Our findings suggest several potential enhancements in nursing bibliometric studies. For instance, in chronological studies, where trends often reveal an increase in article publications across various topics, it is crucial to consider the expanding pool of indexed journals. This consideration ensures an accurate interpretation of trends and minimizes the risk of misinterpretations. Similarly, investigations into document types or languages should account for the indexing policies of scholarly databases like Scopus and Web of Science, which may lead to an overrepresentation of English publications.

Our exploration into authorship and collaboration analyses highlights the prevalent use of full counting, but fractional counting emerges as a superior method for proper field normalization and network construction, thereby enhancing the precision and reliability of results. Moreover, when identifying core journals and applying Bradford's law of scattering, careful consideration of different subject operationalizations is essential, as these choices profoundly influence outcomes.

The challenges surrounding research funding source identification underscore the imperative of integrating multiple sources to ensure comprehensive and accurate analysis, given the inconsistent information across databases. Our analysis also shows the methodological intricacies of determining research topics in nursing literature. We underscore the limitations of journal classifications in capturing specific topics or themes within individual articles, advocating for more flexible approaches such as keyword extraction from titles and abstracts, combined with database‐provided keywords.

We provide examples of twelve bibliometric approaches for the analysis of scholarly outputs in nursing. Possibly, the most surprising gap in nursing bibliometric literature is the absence of gender analyses of authorship in the discipline. Bibliometric studies confirming gender imbalances in research output are prevalent (Larivière et al., 2013), including nursing research (Porter, 2018; Shields et al., 2011). Given that women make up a large majority of members of the profession and the academic discipline of nursing, the lack of further studies on the analysis of gender imbalance among authors in the field is surprising.

### 4.3 Limitations

The scope of our analysis is limited by the coverage of Scopus, which has been criticized for its overrepresentation of English language journals (Mongeon & Paul‐Hus, 2016) and its underrepresentation of journals from the Global South (Borrego et al., 2023). In addition, our search was limited to the presence of the term “bibliometrics” in the titles of the documents, which prevented the retrieval of related concepts such as “citation analysis”, “impact factor” or “scientometrics”, to name just a few. Nevertheless, we consider that the papers surveyed offer a fairly complete overview of the applications of bibliometrics in nursing.

## 5 CONCLUSION

Bibliometric studies have proved useful to map nursing research. These studies have been relevant to quantify the scholarly output in the field, to understand the social structure of the scientific community that engages in knowledge creation, and to assess the maturity of the discipline. The analysis of references and citations has been applied to measure the consumption of scientific information and has proved to be suitable to assess the comprehensiveness of systematic reviews and clinical guidelines. Further research is still needed to explore the coverage of the nursing literature by new bibliographic data sources and to explore topics such as gender imbalance in research, an issue of great relevance in nursing science.

## AUTHOR CONTRIBUTIONS

Belén Mezquita, Ángel Borrego made substantial contributions to conception and design, or acquisition of data, or analysis and interpretation of data. Belén Mezquita, Cristina Alfonso‐Arias, Patricia Martínez‐Jaimez, Ángel Borrego involved in drafting the manuscript or revising it critically for important intellectual content. Belén Mezquita, Cristina Alfonso‐Arias, Patricia Martínez‐Jaimez, Ángel Borrego has given final approval of the version to be published. Each author should have participated sufficiently in the work to take public responsibility for appropriate portions of the content. Belén Mezquita, Ángel Borrego agreed to be accountable for all aspects of the work in ensuring that questions related to the accuracy or integrity of any part of the work are appropriately investigated and resolved.

## 6 FUNDING INFORMATION

This research received no specific grant from any funding agency in the public, commercial, or not‐for‐profit sectors.

## CONFLICT OF INTEREST STATEMENT

The authors have no competing interests.

## ETHICS STATEMENT

No Research Ethics Committee approval was needed.

## Data Availability

The data that support the findings of this study are openly available in Zenodo at https://doi.org/10.5281/zenodo.7928599.

## Nursing bibliometric papers surveyed

### 1

[1] Agra, M. A. C., de Freitas, T. C. S., Caetano, J. Á., Alexandre, A. C. S., Sá, G. G. M., & Neto, N. M. G. (2018). Nursing dissertations and theses on the mobile emergency care services: A bibliometric study. Texto e Contexto Enfermagem, 27(1) doi:10.1590/0104‐07072018003500016

[2] Ai, Y., Sun, K., & Hu, H. (2017). Bibliometric analysis of papers on mild cognitive impairment nursing in China. International Journal of Nursing Sciences, 4(1), 73–79. doi:10.1016/j.ijnss.2016.10.005

[3] Alcalá‐Albert, G. J., & Parra‐González, M. E. (2021). Bibliometric analysis of scientific production on nursing research in the web of science. Education Sciences, 11(9) doi:10.3390/educsci11090455

[4] Anderson, C. A., Keenan, G., & Jones, J. (2009). Using bibliometrics to support your selection of a nursing terminology set. CIN—Computers Informatics Nursing, 27(2), 82–90. doi:10.1097/NCN.0b013e3181972a24

[5] Avena, M. J., & Barbosa, D. A. (2017). Bibliometric indicators of the nursing journals according to the index databases. Revista da Escola de Enfermagem, 51 doi:10.1590/S1980‐220X2017014603262

[6] Backes, V. M., do Prado, M. L., Lino, M. M., Ferraz, F., Canever, B. P., Gomes, D. C., & Martini, J. G. (2013). Theses and dissertations of nurses about education in nursing and health: A bibliometric study. Revista Brasileira de Enfermagem, 66(2), 251–256. doi:10.1590/s0034‐71672013000200015

[7] Baggio, M. A., Rodrigues, M. A., Erdmann, A. L., Figueiredo, M. C. A. B., & Vieira, M. M. S. (2014). Production of nursing thesis and dissertations in Portugal, 2000‐2010: A bibliometric study. Texto e Contexto Enfermagem, 23(2), 250–260. doi:10.1590/0104‐07072014002190012

[8] Ball, E., McLoughlin, M., & Darvill, A. (2011). Plethora or paucity: A systematic search and bibliometric study of the application and design of qualitative methods in nursing research 2008‐2010. Nurse Education Today, 31(3), 299–303. doi:10.1016/j.nedt.2010.12.002

[9] Benton, A. D., Ferguson, S. L., Douglas, J. P., & Benton, D. C. (2021). Contrasting views on scope of practice: A bibliometric analysis of allied health, nursing, and medical literature. Journal of Nursing Regulation, 12(1), 4–18. doi:10.1016/S2155‐8256(21)00016‐8

[10] Benton, D. C., & Brenton, A. S. (2020). Focus and trends in nurse advocacy in the pan American health region: A bibliometric analysis. Revista Latino‐Americana de Enfermagem, 28, 1–8. doi:10.1590/1518‐8345.4368.3312

[11] Beserra, P. J. F., Gomes, G. L. L., Santos, M. C. F., Bittencourt, G. K. G. D., & Nóbrega, M. M. L. D. (2018). Scientific production of the international classification for nursing practice: A bibliometric study. Revista Brasileira de Enfermagem, 71(6), 2860–2868. doi:10.1590/0034‐7167‐2017‐0411

[12] Bilik, O., Damar, H. T., Ozdagoglu, G., Ozdagoglu, A., & Damar, M. (2020). Identifying trends, patterns, and collaborations in nursing career research: A bibliometric snapshot (1980–2017). Collegian, 27(1), 40–48. doi:10.1016/j.colegn.2019.04.005

[13] Blažun Vošner, H., Carter‐Templeton, H., Završnik, J., & Kokol, P. (2020). Nursing informatics: A historical bibliometric analysis. CIN—Computers Informatics Nursing, 38(7), 331–337. doi:10.1097/CIN.0000000000000624

[14] Blažun Vošner, H., Kokol, P., & Vošner, J. (2015). Research literature production on nursing competences from 1981 till 2012: A bibliometric snapshot. Nurse Education Today, 35(5), 673–679. doi:10.1016/j.nedt.2015.01.002

[15] Blažun Vošner, H., Kokol, P., Železnik, D., & Završnik, J. (2019). Identifying historical roots of knowledge development in cardiovascular nursing using bibliometrics. International Journal of Nursing Practice, 25(3) doi:10.1111/ijn.12726, e12726.

[16] Camacho Rodríguez, D. E., Oviedo Córdoba, H. R., Ramos de la Hoz, E., & González Noguera, T. C. (2016). Bibliometric analysis of articles on nursing care published in Colombian magazines. Enfermeria Global, 15(4), 396–405. doi:10.6018/eglobal.15.4.248711.

[17] Cant, R., Cooper, S., & Liaw, S. Y. (2022). An update on the most influential nursing simulation studies: A bibliometric analysis. Clinical Simulation in Nursing, 69, 7–17. doi:10.1016/j.ecns.2022.05.003.

[18] Cant, R., Ryan, C., & Kardong‐Edgren, S. (2022). Virtual simulation studies in nursing education: A bibliometric analysis of the top 100 cited studies, 2021. Nurse Education Today, 114 doi:10.1016/j.nedt.2022.105385, 105385.

[19] Carter‐Templeton, H., Frazier, R. M., Wu, L., & H. Wyatt, T. (2018). Robotics in nursing: A bibliometric analysis. Journal of Nursing Scholarship, 50(6), 582–589. doi:10.1111/jnu.12399.

[20] Chang, C., Gau, M., Tang, K., & Hwang, G. (2021). Directions of the 100 most cited nursing student education research: A bibliometric and co‐citation network analysis. Nurse Education Today, 96 doi:10.1016/j.nedt.2020.104645, 104645.

[21] Cho, I., Kim, D., & Park, H. (2022). Bibliometrics and co‐citation network analysis of systematic reviews of evidence‐based nursing guidelines for preventing inpatient falls. CIN—Computers Informatics Nursing, 40(2), 95–103. doi:10.1097/CIN.0000000000000819.

[22] Çiçek Korkmaz, A., & Altuntaş, S. (2022). A bibliometric analysis of COVID‐19 publications in nursing by visual mapping method. Journal of Nursing Management, 30(6), 1892–1902. doi:10.1111/jonm.13636.

[23] Cullen, J. G. (2016). Nursing management, religion and spirituality: A bibliometric review, a research agenda and implications for practice. Journal of Nursing Management, 24(3), 291–299. doi:10.1111/jonm.12340.

[24] Currie, J., Borst, A. C., & Carter, M. (2022). Bibliometric review of the field of Australian nurse practitioner research between January 2000 to may 2021. Collegian, 29(5), 671–679. doi:10.1016/j.colegn.2022.03.001.

[25] Currie, J., & Chipps, J. (2015). Mapping the field of military nursing research 1990‐2013: A bibliometric review. International Journal of Nursing Studies, 52(10), 1607–1616. doi:10.1016/j.ijnurstu.2015.06.008.

[26] da Silva, A. M. F., Martini, J. G., & Becker, S. G. (2011). The social representation theory in graduate nursing dissertations and theses: A bibliometric profile. Texto e Contexto Enfermagem, 20(2), 294–300. doi:10.1590/S0104‐07072011000200011.

[27] Dardas, L. A., Sawair, F. A., Nabolsi, M., & Simmons, L. A. (2019). Nursing research in the Arab region: A bibliometric analysis. International Journal of Nursing Practice, 25(3) doi:10.1111/ijn.12716, e12716.

[28] De Azevedo, K. C. C., Batista, J. B. V., De Azevedo, R. C., De Araújo, A. L. B., De Oliveira Barros, E., & De Sousa Dantas Rodrigues, M. (2019). National scientific production on burnout syndrome in ICU nurses and physicians: A bibliometric study. Revista Da Associacao Medica Brasileira, 65(5), 722–729. doi:10.1590/1806‐9282.65.5.722.

[29] de Holanda, E. R., Lira, M. C. C., Galvão, M. T. G., Damasceno, M. M. C., & de Araujo, T. L. (2014). Tendencies in the production of scientific knowledge in nursing regarding HIV/AIDS: A bibliometric study. Online Brazilian Journal of Nursing, 12(4), 986–997. doi:10.5935/1676‐4285.20133818.

[30] de Jesus, L. A., Costa, L. E. L., Oliveira, M. G., Dos Santos Souza, V. R., da Silva, G. T. R., Cordeiro, A. L. A. O., & da Silva, R. S. (2022). Nursing consultation teaching in nurses' training: A bibliometric study. Cogitare Enfermagem, 27 doi:10.5380/CE.V27I0.87715, 1–13.

[31] de Oliveira Silva, R. M., de Souza, R. S. A., da Mota, L. S. R., Fernandes, J. D., Souza‐Machado, C., & Oliveira Cordeiro, A. L. A. (2017). Nursing knowledge production on residence: A bibliometric study. Online Brazilian Journal of Nursing, 16(3), 309–318. doi:10.17665/1676‐4285.20175861.

[32] de Oliveira, D. G., Reis, A. D. C., Franco, I. M., & Braga, A. L. (2021). Exploring global research trends in burnout among nursing professionals: A bibliometric analysis. Healthcare, 9(12) doi:10.3390/healthcare9121680.

[33] Dong, J., Wei, W., Wang, C., Fu, Y., Li, Y., Li, J., & Peng, X. (2020). Research trends and hotspots in caregiver studies: A bibliometric and scientometric analysis of nursing journals. Journal of Advanced Nursing, 76(11), 2955–2970. doi:10.1111/jan.14489.

[34] Ergul, S., Ardahan, M., Temel, A. B., & Yildirim, B. Ö. (2010). Bibliometric review of references of nursing research papers during the decade 1994‐2003 in Turkey. International Nursing Review, 57(1), 49–55. doi:10.1111/j.1466‐7657.2009.00770.x.

[35] Estabrooks, C. A., Winther, C., & Derksen, L. (2004). Mapping the field: A bibliometric analysis of the research utilization literature in nursing. Nursing Research, 53(5), 293–303. doi:10.1097/00006199‐200409000‐00003.

[36] Fernandes, G. C. M., Becker, S. G., da Silva Ramos, D. J., do Prado, R. A., dal Sasso, G. M., & Martins, C. R. (2011). Expressions of art in nursing education and care: Bibliometric study. Texto e Contexto Enfermagem, 20(1), 167–174. doi:10.1590/S0104‐07072011000100020.

[37] Ferreira, M. A. L., Pereira, A. M. N. A., Martins, J. C. A., & Barbieri‐Figueiredo, M. C. (2016). Palliative care and nursing in dissertations and theses in Portugal: A bibliometric study. Revista da Escola de Enfermagem, 50(2), 313–319. doi:10.1590/S0080‐623420160000200019.

[38] Ghamgosar, A., Zarghani, M., & Nemati‐Anaraki, L. (2021). Bibliometric analysis on geriatric nursing research in web of science (1900‐2020). BioMed Research International, 2021 doi:10.1155/2021/8758161, 1–11.

[39] Giménez‐Espert, M. D. C., & Prado‐Gascó, V. J. (2019). Bibliometric analysis of six nursing journals from the web of science, 2012–2017. Journal of Advanced Nursing, 75(3), 543–554. doi:10.1111/jan.13868.

[40] Goode, C. J., McCarty, L. B., Fink, R. M., Oman, K. S., Makic, M. F., Krugman, M. E., & Traditi, L. (2013). Mapping the organization: A bibliometric analysis of nurses' contributions to the literature. Journal of Nursing Administration, 43(9), 481–487. doi:10.1097/NNA.0b013e3182a23db5.

[41] Gregorio‐Chaviano, O., Méndez‐Rátiva, C. P., González, M. J. P., & Guzmán, M. F. (2015). Colombian nursing research. A bibliometric analysis of the visibility in ISI WoS (2001‐2013). Enfermeria Global, 14(4), 175–191. doi:10.6018/eglobal.14.4.206751.

[42] Guo, L., Lu, G., & Tian, J. (2020). A bibliometric analysis of cirrhosis nursing research on web of science. Gastroenterology Nursing, 43(3), 232–240. doi:10.1097/SGA.0000000000000457.

[43] Hahn, S., & Ryu, Y. M. (2022). Trends in research on clinical reasoning in nursing over the past 20 years: A bibliometric analysis. Science Editing, 9(2), 112–119. doi:10.6087/kcse.276.

[44] Han, S., Cheng, J., Wang, Y., Chen, Q., & Xu, Z. (2020). A bibliometric analysis of core articles of international nursing research frontiers based on the web of science database. Frontiers of Nursing, 7(1), 31–38. doi:10.2478/fon‐2020‐0010.

[45] Huang, Q., Huang, Q., Liu, Q., Yu, S., & Sun, H. (2022). Tendency and foci of nurse practitioners: Bibliometric analysis based on the CiteSpaceV. Frontiers of Nursing, 9(2), 197–207. doi:10.2478/fon‐2022‐0027.

[46] Huang, Q., Ronghuang, Q., Yinhuang, R., Fanghuang, Y., & Yansun, H. (2021). Trends and hotspots of family nursing research based on web of science: A bibliometric analysis. Japan journal of Nursing Science, 18(2) doi:10.1111/jjns.12401.

[47] Huang, Y., Ho, Y., & Chuang, K. (2006). Bibliometric analysis of nursing research in Taiwan 1991‐2004. Journal of Nursing Research, 14(1), 75–81. doi:10.1097/01.JNR.0000387564.57188.b4.

[48] Hunt, G. E., Jackson, D., Watson, R., & Cleary, M. (2013). A citation analysis of nurse education journals using various bibliometric indicators. Journal of Advanced Nursing, 69(7), 1441–1445. doi:10.1111/jan.12069.

[49] Hutchinson, M., Higson, M., & Jackson, D. (2017). Mapping trends in the concept of nurse rounding: A bibliometric analysis and research agenda. International Journal of Nursing Practice, 23(6) doi:10.1111/ijn.12584.

[50] Imani, B., Mirezati, S. Z., & Saberi, M. K. (2019). A bibliometric analysis of international journal of nursing studies (1963‐2018). Library Philosophy and Practice, digitalcommons.unl.edu/libphilprac/2677/.

[51] Jarden, R. J., Narayanan, A., Sandham, M., Siegert, R. J., & Koziol‐Mclain, J. (2019). Bibliometric mapping of intensive care nurses' wellbeing: Development and application of the new iAnalysis model. BMC Nursing, 18(1) doi:10.1186/s12912‐019‐0343‐1, 21.

[52] Kahwa, E., Dodd, A., Conklin, J. L., Woods Giscombe, C., Leak Bryant, A., Munroe, D., Henry Ferguson, V., Gordon Singh, S., Lynch, M., & Bolton, A. (2022). A bibliometric analysis of nursing and midwifery research in the Caribbean. Journal of Nursing Scholarship, 54(2), 226–233. doi:10.1111/jnu.12721.

[53] Kantek, F., & Yesilbas, H. (2020). Conflict in nursing studies: A bibliometric analysis of the top 100 cited papers. Journal of Advanced Nursing, 76(10), 2531–2546. doi:10.1111/jan.14463.

[54] Kantek, F., Yesilbas, H., & Aytur Ozen, T. (2023). Leadership and care in nursing research: A bibliometric analysis. Journal of Advanced Nursing, 79(3), 1119–1128. doi:10.1111/jan.15527.

[55] Kokol, P. (2021). Meta approaches in knowledge synthesis in nursing: A bibliometric analysis. Nursing Outlook, 69(5), 815–825. doi:10.1016/j.outlook.2021.02.006.

[56] Kokol, P., & Blažun Vošner, H. (2019). Historical, descriptive and exploratory analysis of application of bibliometrics in nursing research. Nursing Outlook, 67(6), 680–695. doi:10.1016/j.outlook.2019.04.009.

[57] Kokol, P., & Blažun Vošner, H. (2017). Nursing informatics research: A bibliometric analysis of funding patterns. Online Journal of Nursing Informatics, 21(2), ojni.org/OJNI‐V21‐N2.pdf.

[58] Kokol, P., Blažun Vošner, H., Vošner, J., & Saranto, K. (2014). Nursing informatics competencies: Bibliometric analysis. Paper Presented at the Studies in Health Technology and Informatics, 201, 342–348. doi:10.3233/978‐1‐61499‐415‐2‐342.

[59] Kokol, P., Železnik, D., Završnik, J., & Blažun Vošner, H. (2019). Nursing research literature production in terms of the scope of country and health determinants: A bibliometric study. Journal of Nursing Scholarship, 51(5), 590–598. doi:10.1111/jnu.12500.

[60] Lanzoni, G. M. M., Baggio, M. A., Parizoto, G. M., Cechinel, C., Erdmann, A. L., Meirelles, B. H. S., & dos Santos, R. N. M. (2011). The grounded theory: A bibliometric study of Brasilian nursing. Index de Enfermeria, 20(3). doi: 10.4321/S1132‐12962011000200015, 209–214.

[61] Lazzari, C., McAleer, S., & Rabottini, M. (2022). The assessment of interprofessional practice in mental health nursing with ethnographic observation and social network analysis: A confirmatory and bibliometric network study using VOSviewer. Rivista di Psichiatria, 57(3), 115–122. doi:10.1708/3814.37989.

[62] Le, Y., Cao, S., Wang, M., Lin, X., & Qian, B. (2021). A bibliometric and visualized analysis of nursing informatics competencies in China (2000–2020) doi:10.3233/SHTI210759.

[63] Lima, M. M., Almeida, A. B., Giovannetti, M. O., Backes, V. M., & Kloh, D. (2012). Knowledge production about nurse education: A bibliometric study. Revista Brasileira de Enfermagem, 65(3), 522–528. doi:10.1590/s0034‐71672012000300019.

[64] Liu, J., Liu, S., Shi, Q., & Wang, M. (2021). Bibliometric analysis of nursing informatics research. Studies in Health Technology and Informatics, doi:10.3233/SHTI210661

[65] Liu, J., Liu, S., Zheng, T., & Fang, J. (2021). Development of Nursing Informatics in Mainland China: A Bibliometric Analysis doi:10.3233/SHTI210664.

[66] Liu, Y., Li, X., Wang, Y., & Li, X. (2022). Dignity in nursing: A bibliometric and visual analysis of scientific publications. Scandinavian Journal of Caring Sciences, doi:10.1111/scs.13118, 37, 384–396.

[67] Lizarbe Chocarro, M. (2007). Nursing in intensive and coronary care. Bibliometric analysis of 180 original articles. Enfermería Intensiva, 18(3), 126–137. doi:10.1016/S1130‐2399(07)74394‐8

[68] Lucena, P. L. C., da Costa, S. F. G., Batista, J. B. V., Lucena, C. M. F., Morais, G. S. N., & Costa, B. H. S. (2018). Scientific production on workplace bullying and nursing: A bibliometric study. Revista da Escola de Enfermagem, 52 doi:10.1590/S1980‐220X2017029103354

[69] Luque‐Alcaraz, O. M., Aparicio‐Martinez, P., Gomera, A., & Vaquero‐Abellán, M. (2022). Nurses as agents for achieving environmentally sustainable health systems: A bibliometric analysis. Journal of Nursing Management, 30(8), 3900–3908. doi:10.1111/jonm.13798

[70] Lyu, L., Li, G., Li, J., & Li, M. (2016). Nurse turnover research in China: A bibliometric analysis from 2000 to 2015. International Journal of Nursing Sciences, 3(2), 208–212. doi:10.1016/j.ijnss.2016.04.008

[71] Marcellus, L. (2019). Bibliometric and textual analysis of historical patterns in maternal‐infant health and nursing issues in the Canadian nurse journal, 1905‐2015. The Canadian Journal of Nursing Research = Revue Canadienne De Recherche En Sciences Infirmieres, 51(2), 53–62. doi:10.1177/0844562118804119.

[72] Martín, A. B. B., Jurado, M. M. M., Pérez‐Fuentes, M. C., Márquez, M. M. S., Sisto, M., & Linares, J. J. G. (2020). Published research on burnout in nursing in Spain in the last decade: Bibliometric analysis. Healthcare, 8(4) doi:10.3390/healthcare8040478

[73] Martínez‐Martínez, C., Esteve‐Claramunt, F., Prieto‐Callejero, B., & Ramos‐Pichardo, J. D. (2022). Stigma towards mental disorders among nursing students and professionals: A bibliometric analysis. International Journal of Environmental Research and Public Health, 19(3) doi:10.3390/ijerph19031839

[74] McVicar, A., Munn‐Giddings, C., & Abu‐Helil, C. (2012). Exploring the development of action research in nursing and social care in the UK: A comparative bibliometric review of action research designs in social work (2000‐2010). Action Research, 10(1), 79–101. doi:10.1177/1476750312439902

[75] Merighi, M. A. B., Gonçalves, R., & Ferreira, F. C. (2007). Bibliometric study on nursing theses and dissertations employing a phenomenological approach: Tendency and perspectives. Revista Latino‐Americana de Enfermagem, 15(4), 645–650. doi:10.1590/S0104‐11692007000400019

[76] Molassiotis, A., Guo, C., Abu‐Odah, H., West, C., & Loke, A. Y. (2021). Evolution of disaster nursing research in the past 30 years (1990–2019): A bibliometric and mapping analysis. International Journal of Disaster Risk Reduction, 58 doi:10.1016/j.ijdrr.2021.102230, 102230.

[77] Mondragón‐Sánchez, E. J., da Costa Pinheiro, P. N., de Paula, P. H. A., da Silva dos Santos, M. H., Ferreira, A. G. N., & Zuluaga, J. E. A. (2022). Qualitative research in nursing: Bibliometric study. Qualitative Report, 27(1), 1–20. doi:10.46743/2160‐3715/2022.5243

[78] Montero, G. H., Díaz, J. D., Pérez, F. P., Hernández, Y. A., Díaz, V. B., & Fragoso, R. H. (2022). Bibliometric analysis of publications about the nursing care process in the period 2015‐2020. Revista Cubana de Enfermería, 38(1), scielo.sld.cu/scielo.php?script=sci_arttext&pid=S0864‐03192022000100004.

[79] Navascués, M. L. J., & Hijar Ordovás, C. A. (2012). Bibliometric study (2001‐2009) on geriatric and gerontologic nursing in Spain. Gerokomos, 23(2), 55–58. doi:10.4321/S1134‐928X2012000200002

[80] Neto, N. M. G., Martins, K., das Neves Silva, P. M., Silva, R. X., Alexandre, A. C. S., & de Moura Sá, G. G. (2020). Scientific production on cardiorespiratory arrest in Brazilian nursing journals: Bibliometric study. Revista Baiana de Enfermagem, 34 doi:10.18471/rbe.v34.36363

[81] Neves, V. R., & Sanna, M. C. (2012). Nursing leadership teaching: A bibliometrics study. ACTA Paulista de Enfermagem, 25(2), 308–313. doi:10.1590/S0103‐21002012000200024

[82] Nicoll, L. H., Carter‐Templeton, H., Oermann, M. H., Ashton, K. S., Edie, A. H., & Conklin, J. L. (2018). A bibliometric analysis of 81 articles that represent excellence in nursing publication. Journal of Advanced Nursing, 74(12), 2894–2903. doi:10.1111/jan.13835

[83] Nicoll, L. H., Oermann, M. H., Carter‐Templeton, H., Owens, J. K., & Edie, A. H. (2020). A bibliometric analysis of articles identified by editors as representing excellence in nursing publication: Replication and extension. Journal of Advanced Nursing, 76(5), 1247–1254. doi:10.1111/jan.14316

[84] Oh, J., & Kim, A. (2020). A bibliometric analysis of COVID‐19 research published in nursing journals. Science Editing, 7(2), 118–124. doi:10.6087/KCSE.205

[85] Pardo, C., Reolid, M., Delicado, M., Mallebrera, E., & García‐Meseguer, M. (2001). Nursing research in Spain: Bibliometrics of references of research papers in the decade 1985‐1994. Journal of Advanced Nursing, 35(6), 933–943. doi:10.1046/j.1365‐2648.2001.01931.x

[86] Peng, J., & Hui, Z. (2011). Nursing research in three regions in China: A bibliometric study. International Nursing Review, 58(1), 21–25. doi:10.1111/j.1466‐7657.2010.00873.x

[87] Peng, K., Mai, Q., Meng, M., Wang, D., Zhang, X., & Hao, Y. (2020). A bibliometric analysis of nursing research in COVID‐19 in China. Journal of Integrative Nursing, 2(3), 116–122. doi:10.4103/jin.jin_32_20

[88] Portugal, M. J., & Rodrigues, M. (2011). Bibliometric data of the journal of nursing Referência. Revista de Enfermagem Referencia, 2011(4), 177–179, www.redalyc.org/articulo.oa?id=388239963007.

[89] Prado‐Gascó, V., Giménez‐Espert, M. C., & De Witte, H. (2021). Job insecurity in nursing: A bibliometric analysis. International Journal of Environmental Research and Public Health, 18(2), 1–13. doi:10.3390/ijerph18020663

[90] Ravelli, A. P. X., Fernandes, G. C. M., Barbosa, S. F. F., Simão, E., dos Santos, S. M. A., & Meirelles, B. H. S. (2009). Knowledge production in nursing and aging: A bibliometric study. Texto e Contexto Enfermagem, 18(3), 506–512. doi: 10.1590/S0104‐07072009000300014

[91] Romero, A., La Hoz, J. D., & González, J. D. (2019). Robots in nursing education: A bibliometric analysis. Paper presented at the Journal of Physics: Conference Series, 1391(1) doi:10.1088/1742‐6596/1391/1/012129, 012129.

[92] Santiago, L. C., & Carlantonio, L. F. M. (2015). The production of knowledge in nursing in the BRIC countries: A bibliometric study. Texto e Contexto Enfermagem, 24(2), 486–493. doi:10.1590/0104‐07072015001362014

[93] Scott, S. D., Profetto‐McGrath, J., Estabrooks, C. A., Winther, C., Wallin, L., & Lavis, J. N. (2010). Mapping the knowledge utilization field in nursing from 1945 to 2004: A bibliometric analysis. Worldviews on Evidence‐Based Nursing, 7(4), 226–237. doi:10.1111/j.1741‐6787.2010.00197.x

[94] Shi, J., Wei, S., Gao, Y., Mei, F., Tian, J., Zhao, Y., & Li, Z. (2022). Global output on artificial intelligence in the field of nursing: A bibliometric analysis and science mapping. Journal of Nursing Scholarship, doi:10.1111/jnu.12852

[95] Shi, X., Zhou, Y., & Li, Z. (2022). Bibliometric analysis of the doctor of nursing practice dissertations in the ProQuest dissertations and theses database. Journal of Advanced Nursing, 78(3), 776–786. doi:10.1111/jan.15006

[96] Singh, S., & Pandita, R. (2018). Measurement of global nursing research output: A bibliometric study (1996‐2015). Journal of Information Science Theory and Practice, 6(1), 31–44. doi:10.1633/JISTaP.2018.6.1.3

[97] Smith, D. R. (2010). A longitudinal analysis of bibliometric and impact factor trends among the core international journals of nursing, 1977‐2008. International Journal of Nursing Studies, 47(12), 1491–1499. doi:10.1016/j.ijnurstu.2010.05.006

[98] Sousa, M. H. A., & Figueiredo, A. S. (2021). Nursing records in Portuguese journals (1958‐1998): A bibliometric study. Revista de Enfermagem Referencia, 2021(8) doi:10.12707/RV20173

[99] Su, W., Hwang, G., & Chang, C. (2022). Bibliometric analysis of core competencies associated nursing management publications. Journal of Nursing Management, 30(7), 2869–2880. doi:10.1111/jonm.13795

[100] Sweileh, W. M. (2022). Patient satisfaction with nursing care: A bibliometric and visualization analysis (1950–2021). International Journal of Nursing Practice, 28(5) doi:10.1111/ijn.13076, e13076.

[101] Taffner, V. B. M., Pimentel, R. R. D. S., Almeida, D. B., Freitas, G. F., & Santos, M. J. D. (2022). Nursing theories and models as theoretical references for Brazilian theses and dissertations: A bibliometric study. Revista Brasileira de Enfermagem, 75(4), e20210201. doi:10.1590/0034‐7167‐2021‐0201

[102] Ling TA, Jianni QU, Hong GU, Mei WA, Nini XU, Xiaoyan BA, Xueyan FA, Zhixuan XU, Yihui TI, Fen ZH. (2022). Bibliometrics and visual analysis for clinical research on intervention of traditional Chinese medicine nursing technology on rheumatoid arthritis. Journal of Integrative Nursing, 4(1), 20–25. doi:10.4103/jin.jin_53_21

[103] Taşkaya, S., & Aksoy, A. (2021). A bibliometric analysis of workplace incivility in nursing. Journal of Nursing Management, 29(3), 518–525. doi:10.1111/jonm.13161

[104] Thelwall, M., & Mas‐Bleda, A. (2020). How does nursing research differ internationally? A bibliometric analysis of six countries. International Journal of Nursing Practice, 26(6) doi:10.1111/ijn.12851, e12851.

[105] Traynor, M., Rafferty, A. M., & Lewison, G. (2001). Endogenous and exogenous research? Findings from a bibliometric study of UK nursing research. Journal of Advanced Nursing, 34(2), 212–222. doi:10.1046/j.1365‐2648.2001.01747.x

[106] Tuppal, C. P., Gallardo‐Ninobla, M. M., Arquiza, G. S., & Vega, P. D. (2019). A bibliometric analysis of the Philippine Journal of Nursing for 1966–2017. Philippine Journal of Nursing, 89(1), 32–40, https://drive.google.com/file/d/1N11EJ5c7cfMGqzbzfBS9bCimU9nuSH0u/view.

[107] Unal, A., & Teskereci, G. (2022). Mapping the evidence‐based practice research field in nursing from 1995 to 2021: A bibliometric analysis. International Journal of Nursing Knowledge, 33(3), 196–206. doi:10.1111/2047‐3095.12347

[108] Waldrop, J., Carter‐Templeton, H., & Nicoll, L. H. (2020). Bibliometric impact analysis for the journal for nurse practitioners: 2015‐2017. CIN—Computers Informatics Nursing, 38(1), 1–4. doi:10.1097/CIN.0000000000000613

[109] Wang, C., Shi, Y., Lu, H., Dong, X., Hou, L., Wang, L., Wan, Q., Hu, L., Zhang, L., Dou, D., & Shang, S. (2022). Global nursing research activity from 2009 to 2020: A bibliometric analysis. International Journal of Nursing Practice, 28(5) doi:10.1111/ijn.13063, e13063.

[110] Wang, J., Chen, Y., Zhai, X., Chu, Y., Liu, X., & Ma, X. (2022). Visualizing research trends and identifying hotspots of traditional Chinese medicine (TCM) nursing technology for insomnia: A 18‐years bibliometric analysis of web of science core collection. Frontiers in Neurology, 13 doi:10.3389/fneur.2022.816031

[111] Wang, Y., Li, X., Liu, Y., & Shi, B. (2022). Mapping the research hotspots and theme trends of simulation in nursing education: A bibliometric analysis from 2005 to 2019. Nurse Education Today, 116 doi:10.1016/j.nedt.2022.105426, 105426.

[112] Wiles, L., Olds, T., & Williams, M. (2013). Twenty‐five years of Australian nursing and allied health professional journals: Bibliometric analysis from 1985 through 2010. Scientometrics, 94(1), 359–378. doi:10.1007/s11192‐012‐0704‐y

[113] Williams, J. K., Tripp‐Reimer, T., Daack‐Hirsch, S., & Deberg, J. (2016). Five‐year bibliometric review of genomic nursing science research. Journal of Nursing Scholarship, 48(2), 179–186. doi:10.1111/jnu.12196

[114] Winters, J. R. D. F., Prado, M. L. D., Lazzari, D. D., & Jardim, V. L. T. (2018). Nursing higher education in MERCOSUR: A bibliometric study. Revista Brasileira de Enfermagem, 71, 1732–1739. doi:10.1590/0034‐7167‐2017‐0405

[115] Woods, S., Phillips, K., & Dudash, A. (2020). Grey literature citations in top nursing journals: A bibliometric study. Journal of the Medical Library Association, 108(2), 262–269. doi:10.5195/jmla.2020.760

[116] Woods, S. W., Phillips, K. P., & Dudash, A. D. (2020). Dissertations and theses in top nursing publications: A bibliometric study. Evidence Based Library and Information Practice, 15(4), 68–82. doi:10.18438/eblip29764

[117] Xiao, Q., Wang, J., Wang, Y., & Wu, Y. (2019). Data mining in nursing: A bibliometric analysis (1990–2017) doi:10.3233/SHTI190562

[118] Yanbing, S., Ruifang, Z., Chen, W., Shifan, H., Hua, L., & Zhiguang, D. (2020). Bibliometric analysis of journal of nursing management from 1993 to 2018. Journal of Nursing Management, 28(2), 317–331. doi:10.1111/jonm.12925

[119] Yang, L., Tu, S., Feng, L., Lai, X., & Wang, R. (2021). Bibliometric analysis of multiple sclerosis nursing research based on web of science. Annals of Palliative Medicine, 10(7), 7551–7559. doi:10.21037/apm‐21‐1057

[120] Yesilbas, H., & Kantek, F. (2022). Trends and hot topics in nurse empowerment research: A bibliometric analysis. Japan Journal of Nursing Science, 19(2) doi:10.1111/jjns.12458

[121] Železnik, D., Blažun Vošner, H., & Kokol, P. (2017). A bibliometric analysis of the journal of advanced nursing, 1976–2015. Journal of Advanced Nursing, 73(10), 2407–2419. doi:10.1111/jan.13296

[122] Zhang, Q., Chen, J., & Liu, J. (2022). Global trends and hot‐spots in research on virtual simulation in nursing: A bibliometric analysis from 1999 to 2021. Frontiers in Public Health, 10 doi:10.3389/fpubh.2022.890773

[123] Zhang, Q., Li, S., Liu, J., & Chen, J. (2022). Global trends in nursing‐related research on COVID‐19: A bibliometric analysis. Frontiers in Public Health, 10, 933555. doi:10.3389/fpubh.2022.933555

[124] Zhang, X., Huang, D., & Li, F. (2011). Cancer nursing research output and topics in the first decade of the 21st century: Results of a bibliometric and co‐word cluster analysis. Asian Pacific Journal of Cancer Prevention, 12(8), 2055‐2058, journal.waocp.org/article_25836.html.

[125] Zhang, Y., Zhu, L., Sheng, Y., Li, X., Xu, X., & Wang, Q. (2018). Disaster nursing development in China and other countries: A bibliometric study. Journal of Nursing Scholarship, 50(5), 567–576. doi:10.1111/jnu.12401

[126] Zhao, J., Liu, X., Zhang, W., Xing, Y., Cho, S. W., & Hao, Y. (2018). Evidence‐based nursing outputs and hot spot analysis of the last 5 years in mainland China: Results of a bibliometric analysis. International Journal of Nursing Practice, 24(2) doi:10.1111/ijn.12628, e12628.

[127] Zhao, J., Lu, Y., Zhou, F., Mao, R., & Fei, F. (2022). Systematic bibliometric analysis of research hotspots and trends on the application of virtual reality in nursing. Frontiers in Public Health, 10 doi:10.3389/fpubh.2022.906715

[128] Zhu, R., Liu, M., Su, Y., Meng, X., Han, S., & Duan, Z. (2021). A bibliometric analysis of publication of funded studies in nursing research from web of science, 2008–2018. Journal of Advanced Nursing, 77(1), 176–188. doi:10.1111/jan.14578

[129] Zhu, R., Wang, Y., Wu, R., Meng, X., Han, S., & Duan, Z. (2020). Trends in high‐impact papers in nursing research published from 2008 to 2018: A web of science‐based bibliometric analysis. Journal of Nursing Management, 28(5), 1041–1052. doi:10.1111/jonm.13038

## References

[nop270036-bib-0001] Alcalá‐Albert, G. J. , & Parra‐González, M. E. (2021). Bibliometric analysis of scientific production on nursing research in the web of science. Educational Sciences, 11(9), 455. 10.3390/educsci11090455

[nop270036-bib-0002] Alfonzo, P. M. , Sakraida, T. J. , & Hastings‐Tolsma, M. (2014). Bibliometrics: Visualizing the impact of nursing research. Online Journal of Nursing Informatics, 18(1), 18–33. https://ojni.org/archive.html.

[nop270036-bib-0004] Borrego, Á. , Ardanuy, J. , & Arguimbau, L. (2023). Crossref as a bibliographic discovery tool in the arts and humanities. Quantitative Science Studies, 4(1), 91–104. 10.1162/qss_a_00240

[nop270036-bib-0005] Borrego, Á. , & Mezquita, B. (2023). Data for "the use of bibliometrics in nursing research: Topics, data sources and applications" [data set]. Zenodo. 10.5281/zenodo.7928599

[nop270036-bib-0006] Cant, R. , Ryan, C. , & Kardong‐Edgren, S. (2022). Virtual simulation studies in nursing education: A bibliometric analysis of the top 100 cited studies. Nurse Education Today, 114, 105385. 10.1016/j.nedt.2022.105385 35569265

[nop270036-bib-0007] Chawla, D. S. (2022). Massive open index of scholarly papers launches. Nature News. 10.1038/d41586-022-00138-y 35075274

[nop270036-bib-0008] Davidson, P. M. , Newton, P. J. , Ferguson, C. , Daly, J. , Elliott, D. , Homer, C. , Duffield, C. , & Jackson, D. (2014). Rating and ranking the role of bibliometrics and webometrics in nursing and midwifery. The Scientific World Journal, 135812. 10.1155/2014/135812 24550691 PMC3914409

[nop270036-bib-0009] De Groote, S. L. , & Raszewski, R. (2012). Coverage of Google scholar, Scopus, and web of science: A case study of the h‐index in nursing. Nursing Outlook, 60(6), 391–400. 10.1016/j.outlook.2012.04.007 22748758

[nop270036-bib-0010] Desai, N. , Veras, L. , & Gosain, A. (2018). Using Bradford's law of scattering to identify the core journals of pediatric surgery. Journal of Surgical Research, 229, 90–95. 10.1016/j.jss.2018.03.062 29937022

[nop270036-bib-0011] Donthu, N. , Kumar, S. , Mukherjee, D. , Pandey, N. , & Lim, W. M. (2021). How to conduct a bibliometric analysis: An overview and guidelines. Journal of Business Research, 133, 285–296. 10.1016/j.jbusres.2021.04.070

[nop270036-bib-0012] Gusenbauer, M. (2022). Search where you will find most: Comparing the disciplinary coverage of 56 bibliographic databases. Scientometrics, 127(5), 2683–2745. 10.1007/s11192-022-04289-7 35571007 PMC9075928

[nop270036-bib-0013] Gusenbauer, M. , & Haddaway, N. R. (2020). Which academic search systems are suitable for systematic reviews or meta‐analyses? Evaluating retrieval qualities of Google scholar, PubMed, and 26 other resources. Research Synthesis Methods, 11(2), 181–217. 10.1002/jrsm.1378 31614060 PMC7079055

[nop270036-bib-0014] Harzing, A. W. (2019). Two new kids on the block: How do Crossref and dimensions compare with Google scholar, Microsoft academic, Scopus and the web of science? Scientometrics, 120(1), 341–349. 10.1007/s11192-019-03114-y

[nop270036-bib-0016] Kokol, P. (2023). Discrepancies among Scopus and web of science, coverage of funding information in medical journal articles: A follow‐up study. Journal of the Medical Library Association, 111(3), 703–709. 10.5195/jmla.2023.1513 37483361 PMC10361553

[nop270036-bib-0017] Kokol, P. , & Vošner, H. B. (2018). Discrepancies among Scopus, web of science, and PubMed coverage of funding information in medical journal articles. Journal of the Medical Library Association, 106(1), 81–86. 10.5195/jmla.2018.181 29339937 PMC5764597

[nop270036-bib-0018] Koo, M. , & Lin, S. C. (2023). An analysis of reporting practices in the top 100 cited health and medicine‐related bibliometric studies from 2019 to 2021 based on a proposed guidelines. Heliyon, 9(6), e16780. 10.1016/j.heliyon.2023.e16780 37292336 PMC10245063

[nop270036-bib-0019] Larivière, V. , Ni, C. , Gingras, Y. , Cronin, B. , & Sugimoto, C. R. (2013). Bibliometrics: Global gender disparities in science. Nature, 504(7479), 211–213. 10.1038/504211a 24350369

[nop270036-bib-0020] Martín‐Martín, A. , Thelwall, M. , Orduna‐Malea, E. , & Delgado López‐Cózar, E. (2021). Google scholar, Microsoft academic, Scopus, dimensions, web of science, and OpenCitations' COCI: A multidisciplinary comparison of coverage via citations. Scientometrics, 126(1), 871–906. 10.1007/s11192-020-03690-4 32981987 PMC7505221

[nop270036-bib-0021] Marx, W. , Bornmann, L. , Barth, A. , & Leydesdorff, L. (2014). Detecting the historical roots of research fields by reference publication year spectroscopy (RPYS). Journal of the Association for Information Science and Technology, 65(4), 751–764. 10.1002/asi.23089

[nop270036-bib-0022] Mongeon, P. , & Paul‐Hus, A. (2016). The journal coverage of web of science and Scopus: A comparative analysis. Scientometrics, 106(1), 213–228. 10.1007/s11192-015-1765-5

[nop270036-bib-0023] Montazeri, A. , Mohammadi, S. , Hesari, P. , Ghaemi, M. , Riazi, H. , & Sheikhi‐Mobarakeh, Z. (2023). Preliminary guideline for reporting bibliometric reviews of the biomedical literature (BIBLIO): A minimum requirements. Systematic Reviews, 12, 239. 10.1186/s13643-023-02410-2 38102710 PMC10722750

[nop270036-bib-0024] Mukherjee, D. , Lim, W. M. , Kumar, S. , & Donthu, N. (2022). Guidelines for advancing theory and practice through bibliometric research. Journal of Business Research, 148, 101–115. 10.1016/j.jbusres.2022.04.042

[nop270036-bib-0025] Nicolaisen, J. , & Hjørland, B. (2007). Practical potentials of Bradford's law: A critical examination of the received view. Journal of Documentation, 63(3), 359–377. 10.1108/00220410710743298

[nop270036-bib-0026] Pao, M. L. (1985). Lotka's law: A testing procedure. Information Processing & Management, 21(4), 305–320. 10.1016/0306-4573(85)90055-X

[nop270036-bib-0027] Perianes‐Rodriguez, A. , Waltman, L. , & Van Eck, N. J. (2016). Constructing bibliometric networks: A comparison between full and fractional counting. Journal of Informetrics, 10(4), 1178–1195. 10.1016/j.joi.2016.10.006

[nop270036-bib-0028] Porter, S. (2018). Gender and publishing in nursing: A secondary analysis of h‐index ranking tables. Journal of Advanced Nursing, 74(8), 1899–1907. 10.1111/jan.13703 29797603

[nop270036-bib-0029] Powell, K. R. , & Peterson, S. R. (2017). Coverage and quality: A comparison of web of science and Scopus databases for reporting faculty nursing publication metrics. Nursing Outlook, 65(5), 572–578. 10.1016/j.outlook.2017.03.004 28377037

[nop270036-bib-0031] Shields, L. , Hall, J. , & Mamun, A. A. (2011). The ‘gender gap’ in authorship in nursing literature. Journal of the Royal Society of Medicine, 104(11), 457–464. 10.1258/jrsm.2011.110015 22048677 PMC3206719

[nop270036-bib-0032] Smith, D. R. , & Hazelton, M. (2008). Bibliometrics, citation indexing, and the journals of nursing. Nursing & Health Sciences, 10(4), 260–265. 10.1111/j.1442-2018.2008.00414.x 19128301

[nop270036-bib-0033] Smith, D. R. , & Hazelton, M. (2011). Bibliometric awareness in nursing scholarship: Can we afford to ignore it any longer? Nursing & Health Sciences, 13(4), 384–387. 10.1111/j.1442-2018.2011.00652.x 22098385

[nop270036-bib-0034] Smith, D. R. , & Watson, R. (2016). Career development tips for today's nursing academic: Bibliometrics, altmetrics and social media. Journal of Advanced Nursing, 72(11), 2654–2661. 10.1111/jan.13067 27399604

[nop270036-bib-0035] Thompson, D. F. , & Walker, C. K. (2015). A descriptive and historical review of bibliometrics with applications to medical sciences. Pharmacotherapy, 35(6), 551–559. 10.1002/phar.1586 25940769

[nop270036-bib-0036] Van Eck, N. J. , & Waltman, L. (2010). Software survey: VOSviewer, a computer program for bibliometric mapping. Scientometrics, 84(2), 523–538. 10.1007/s11192-009-0146-3 20585380 PMC2883932

[nop270036-bib-0037] Van Raan, A. F. (2004). Sleeping beauties in science. Scientometrics, 59(3), 467–472. 10.1023/B:SCIE.0000018543.82441.f1

[nop270036-bib-0038] Waltman, L. , & van Eck, N. J. (2015). Field‐normalized citation impact indicators and the choice of an appropriate counting method. Journal of Informetrics, 9(4), 872–894. 10.1016/j.joi.2015.08.001

[nop270036-bib-0039] Wang, C. , Shi, Y. , Lu, H. , Dong, X. , Hou, L. , Wang, L. , Wan, Q. , Hu, L. , Zhang, L. , Dou, D. , & Shang, S. (2022). Global nursing research activity from 2009 to 2020: A bibliometric analysis. International Journal of Nursing Practice, 28(5), e13063. 10.1111/ijn.13063 35599432

